# Identification of four novel loci associated with psychotropic drug-induced weight gain in a Swiss psychiatric longitudinal study: A GWAS analysis

**DOI:** 10.1038/s41380-023-02082-3

**Published:** 2023-05-12

**Authors:** Jennifer Sjaarda, Aurélie Delacrétaz, Céline Dubath, Nermine Laaboub, Marianna Piras, Claire Grosu, Frederik Vandenberghe, Séverine Crettol, Nicolas Ansermot, Franziska Gamma, Kerstin Jessica Plessen, Armin von Gunten, Philippe Conus, Zoltan Kutalik, Chin B. Eap

**Affiliations:** 1 Unit of Pharmacogenetics and Clinical Psychopharmacology, Centre for Psychiatric Neuroscience, Department of Psychiatry, Lausanne University Hospital, Prilly, Switzerland; 2 Swiss Institute of Bioinformatics, Lausanne, Switzerland; 3 Les Toises Psychiatry and Psychotherapy Center, Lausanne, Switzerland; 4 Service of Child and Adolescent Psychiatry, Department of Psychiatry, Lausanne University Hospital, University of Lausanne, Prilly, Switzerland; 5 Service of Old Age Psychiatry, Department of Psychiatry, Lausanne University Hospital, Prilly, Switzerland; 6 Service of General Psychiatry, Department of Psychiatry, Lausanne University Hospital, Prilly, Switzerland; 7 University Center for Primary Care and Public Health, University of Lausanne, Lausanne, Switzerland; 8 Department of Computational Biology, University of Lausanne, Lausanne, Switzerland; 9 School of Pharmaceutical Sciences, University of Geneva, University of Lausanne, Geneva, Switzerland; 10 Center for Research and Innovation in Clinical Pharmaceutical Sciences, University of Lausanne, Lausanne, Switzerland; 11 Institute of Pharmaceutical Sciences of Western Switzerland, University of Geneva, University of Lausanne, Geneva, Switzerland

**Keywords:** Genetics, Psychiatric disorders

## Abstract

Patients suffering from mental disorders are at high risk of developing cardiovascular diseases, leading to a reduction in life expectancy. Genetic variants can display greater influence on cardiometabolic features in psychiatric cohorts compared to the general population. The difference is possibly due to an intricate interaction between the mental disorder or the medications used to treat it and metabolic regulations. Previous genome wide association studies (GWAS) on antipsychotic-induced weight gain included a low number of participants and/or were restricted to patients taking one specific antipsychotic. We conducted a GWAS of the evolution of body mass index (BMI) during early (i.e., ≤ 6) months of treatment with psychotropic medications inducing metabolic disturbances (i.e., antipsychotics, mood stabilizers and some antidepressants) in 1135 patients from the PsyMetab cohort. Six highly correlated BMI phenotypes (i.e., BMI change and BMI slope after distinct durations of psychotropic treatment) were considered in the analyses. Our results showed that four novel loci were associated with altered BMI upon treatment at genome-wide significance (*p* < 5 × 10^−8^): rs7736552 (near *MAN2A1*), rs11074029 (in *SLCO3A1*), rs117496040 (near *DEFB1*) and rs7647863 (in *IQSEC1*). Associations between the four loci and alternative BMI-change phenotypes showed consistent effects. Replication analyses in 1622 UK Biobank participants under psychotropic treatment showed a consistent association between rs7736552 and BMI slope (*p* = 0.017). These findings provide new insights into metabolic side effects induced by psychotropic drugs and underline the need for future studies to replicate these associations in larger cohorts.

## Introduction

Compared to the general population, individuals suffering from schizophrenia, bipolar disorder and major depressive disorders have a reduced life expectancy of 10–15 years, which is mainly attributable to natural causes including cardiovascular diseases [1, 2]. Multiple risk factors including genetic and environmental susceptibilities (e.g., mental disorder-related factors, such as an unhealthy lifestyle and/or adverse effects of treatment) may explain the observed excessive propensity of patients suffering from mental disorder for developing metabolic diseases [3, 4]. For instance, the use of psychotropic medications, such as antipsychotics (most atypical but also some typical), mood stabilizers (e.g., lithium), and some antidepressants (e.g., mirtazapine) can increase the risk of metabolic disorders, including obesity through various mechanisms [5, 6]. Among the most widespread mechanistic hypotheses, metabolic side effects induced by psychotropic drugs may result from an appetite increase driven in part by pharmacodynamic factors and modifications in the transcription of some hormones involved in energy homeostasis [7, 8]. Over the last twenty years, pharmacogenetics of psychotropic drug induced weight gain has been extensively studied using candidate gene approaches, in particular within dopamine and serotonin receptors [9, 10]. In addition, numerous single nucleotide polymorphisms (SNPs) within genes involved in other metabolic pathways (e.g., in enzymes, receptors, or transcriptional coactivators involved in food intake homeostasis) were also associated with weight gain in patients with a mental disorder receiving psychotropic drugs [11, 12]. With the emergence of genome-wide association studies (GWAS) conducted in the general population, many additional genetic variants associated with metabolic outcomes such as obesity have been discovered [13, 14]. In particular, this has allowed for the discovery of previously unsuspected genetic variants, such as on the *fat mass and obesity* (*FTO)* gene [15]. However, even though GWAS have been a rich source to provide novel candidate genes and pathways, the explained variance by genetics on cardiometabolic phenotypes remains very low [13, 16]. Thus, it appears that the majority of obesity heritability is still unexplained, suggesting that many genetic variants remain to be determined while taking into account the interaction with environmental factors. In particular, a recent review on the genetic overlap between psychiatric disorders and cardiometabolic diseases emphasized the importance of considering gene-environment interactions in the shared pathways between these diseases [17]. Thus, it has been observed that the psychiatric population displays a greater influence of some genetic variants on metabolic features in comparison to the general population, possibly because of an intricate interaction between the mental disorder or the medications used to treat it and metabolic regulation [18], as well as a higher prevalence of metabolic abnormalities in this specific population [19].

Over the last decade, GWAS have identified only a few genetic loci associated with antipsychotic-induced weight gain [20–24]. These studies included a low number of participants and/or were restricted to patients taking one specific antipsychotic. Considering the previously mentioned aspects, it is of great interest to conduct a GWAS on obesity and/or weight gain in a larger sample of patients with a mental disorder who receive psychotropic drugs inducing metabolic disturbances. This would improve our understanding on biological processes underlying psychotropic drug-induced weight gain.

## Methods

### PsyMetab cohort

Since 2007, a longitudinal observational study is ongoing in the Department of Psychiatry of the Lausanne University Hospital and in a private mental health care center (Les Toises, Lausanne, Switzerland) (PsyMetab) as described elsewhere [25]. Briefly, patients starting a psychotropic treatment associated with a risk of developing metabolic disturbances (i.e. antipsychotics, mood stabilizers or some antidepressants, as listed in the Supplementary Table 1) were included. Clinical data were collected during hospitalization or in outpatient centers during a medical examination based on the department guideline for the metabolic follow-up of psychotropic drugs performed on a routine basis [26]. Monitoring for physical health risk factors include prospective assessments of metabolic factors such as body mass index (BMI) during treatment [27]. When a treatment was stopped for more than two weeks or if a psychotropic drug was replaced by another, the follow-up was restarted from baseline. The present study included patients with informed consent and was approved by the Ethic Committee of Vaud (CER-VD).

### Genotyping and quality control

All participants were genotyped on the Global Screening Array (GSA) v2 with multiple disease option (MD) chip at the Genomics Platform of iGE3 in Geneva, Switzerland (http://www.ige3.unige.ch/genomics-platform.php). All quality control (QC) and filtering steps were performed in PLINK [28] (additional information in Supplementary Information). Only individuals from European ancestry were considered in the present study (Supplementary Information, Supplementary Fig. 1). Genotypes were called using data-driven clustering according to GenomeStudio recommendations, and subsequently exported in PLINK format [28, 29]. Mitochondrial and Y-chromosome variants were removed from downstream data processing and analysis. SNPs were then all aligned to the positive strand and SNPs with MAF of 0 were removed. Standard quality control (QC) filters were applied before imputation (additional information in Supplementary Information) [30]. Further QC filters were applied post-imputation. KING kinship statistics were used to estimate relatedness. Using a kinship threshold of 0.0884 (as recommended by the KING authors), related individuals were removed such that a maximal set of unrelated individuals were retained [31].

Among the retained samples, SNPs were removed if there was significant deviation from Hardy-Weinberg Equilibrium (*p* < 10^−8^). Lastly, SNPs whose minor allele frequency (MAF) was lower than 1% were removed from the dataset.

### Study sample and construction of BMI phenotypes

Only participants with available height, weight and date of follow-up were included. For each participant, height was averaged across all measures to account for variability in measurement. As a quality control measure, participants with height + /− 5 SD outside the mean or with discordant sexes between follow-ups were removed.

Six distinct BMI phenotypes were considered to describe the evolution of BMI in the present study: BMI change (ΔBMI) was defined as the raw change between first and last date of follow-up. We also examined BMI changes after 1-, 3- and 6-months (corresponding to up to 30, 90, and 180 days, respectively) as additional outcomes. Further, BMI slope (βBMI) was defined as the beta coefficient of a linear model between BMI (y) and days of follow-up (x). Sixth, BMI slope was also defined restricting to follow-ups within the first 6 months only. It is noteworthy that there were not enough follow-up measures within 1 or 3 months to create such measures for these periods. To ensure correctly calibrated P-values, all phenotypes were inverse normal quantile transformed (INQT) before performing downstream analyses [32].

### Choice of primary measure

Evolution of BMI throughout psychotropic treatment was chosen as the primary measure because it is a well-known adverse side effect of psychiatric medications. Evolution of BMI evaluated both as a change over time (slope) and raw change (difference between BMI before and after treatment), considering various periods of follow-up (1-, 3- and 6- month) were all considered as primary endpoints. Weight-inducing effect is strongest between 1 and 3 months of treatment, thus various follow-up periods were evaluated. Additionally, BMI and BMI evolution represents a general marker of metabolic health, which is known to be worse in psychiatric cohorts. It is noteworthy that the evolution of additional cardiometabolic risk factors such as waist-to-hip ratio or waist-to-height ratio would also have been valuable. However, waist circumference was recorded only partially in the present study and its consideration would have drastically decreased the power of the study.

### Statistical analyses

The six above-mentioned outcome measures of BMI change were adjusted for the first 20 principal components (PCs), sex, age at first drug administration, the square of this age (age^2^), follow-up time (days), follow-up time squared (days^2^) and BMI at first drug administration (kg/m^2^). In a first step, GWAS association analyses between SNPs and the six phenotypes of BMI evolution were conducted in the whole PsyMetab sample.

Because of an important variability in the risk to induce weight gain across psychotropic drugs (i.e. olanzapine, clozapine and valproate having the highest propensity to induce weight gain, whereas amitriptyline, levomepromazine, lithium, mirtazapine, paliperidone, risperidone, quetiapine, trimipramine and zuclopenthixol are associated with a moderate risk and amisulpride, aripiprazole, carbamazepine, chlorprothixene, flupentixol, haloperidol, lurasidone and tiapride having lower risk to induce weight gain [33]), association analysis between SNPs and the six phenotypes of BMI evolution were also stratified for groups of patients taking the same drug. Of note, patients taking infrequently prescribed drugs (i.e., amitriptyline, levomepromazine, lithium, trimipramine, zuclopenthixol, carbamazepine, chlorprothixene, flupentixol, haloperidol, lurasidone and triapride) were considered in an “other” category). This resulted in 6 (highly-correlated obesity outcomes) x 9 (drugs) genome-wide (GW) association scans.

Adjusted phenotype values were tested for association with SNPs by linear regression in PLINK [28]. A standard GW-significance threshold was applied (5 ×10^−8^) to correct for the approximately 1 million independent statistical tests corresponding to all common genetic variation of the human genome. Only the most associated SNP per locus was kept. Where appropriate (i.e., for aggregating analyses conducted on each drug separately), meta-analyses were performed in PLINK. Finally, meta-analysis results were examined for evidence of heterogeneity to assess if any significant results could be driven by one drug in particular.

### Replication of novel PsyMetab weight-gain associated loci in the UK Biobank (UKB)

We tested the identified GW-significant signals for replication in the UK Biobank (UKB), a large population-based cohort from the UK with rich genotype and phenotype information [34]. We selected a subset of UKB participants to match the most important characteristics of the PsyMetab cohort. Specifically, we selected participants who indicate they were taking a psychotropic drug and/or participants who have been diagnosed with a mental disorder (more details in Supplementary Information).

Only British, unrelated, consenting participants with at least two available BMI measures were included. BMI slope was calculated for each participant as the beta coefficient of the regression of BMI versus time in days (where the first observation was at time 0, considered as “baseline BMI”, and all subsequent measures as days since the first observation). BMI slope was then transformed as PsyMetab with INQT to ensure that data were normally distributed. As with analyses conducted in PsyMetab, BMI slope was adjusted for relevant covariates via residualization for age, sex, baseline BMI and the first 40 genetic principal components. GW-significant SNPs identified in PsyMetab were then tested for an effect on the residualized BMI slope values in participants diagnosed with a mental disorder and/or taking a psychotropic drug from the UKB.

### Use of UKB as a non-psychiatric, population-based sample

We also used the UKB as a non-psychiatric, population-based population, referred to as “UKB population-based sample” in order to determine whether the observed associations are unique to the population of subjects who suffer from a mental disorder or are also associated with the evolution of BMI in the general population. The same participant filters were applied to the UKB as above. Participants who received a psychotropic drug or participants who have been diagnosed with a mental disorder were excluded from the sample. BMI slope was computed as described above, rank-based INQT, and subsequently residualized for age, sex, PC1-40, and baseline BMI. As before, GW-significant SNPs in the PsyMetab sample were assessed for an effect on the residualized BMI slope in the UKB population-based sample.

### Association of loci with metabolic and psychiatric outcomes in consortia and in GeneAtlas

GW-significant SNPs were tested for association with metabolic or psychiatric phenotypes in relevant publicly available consortia (i.e. DIAbetes Genetics Replication And Meta-analysis (DIAGRAM), Genetic Investigation of Anthropometric Traits (GIANT), Meta-Analyses of Glucose and Insulin-related traits Consortium (MAGIC), Psychiatric Genomics Consortium (PGC) and Global Lipids Genetics Consortium (GLGC), listed in Supplementary Table 2), including bipolar disorder (BIP), major depressive disorder (MDD), schizophrenia (SCZ), BMI, type 2 diabetes (T2D), fasting glucose, high-density lipoprotein cholesterol (HDL-cholesterol), low-density lipoprotein cholesterol (LDL-cholesterol), total cholesterol (TC) and triglycerides (TG) [13, 35–40]. Additionally, GW-significant SNPs were also examined for associations with a broad atlas of phenotypes in the UKB using the publicly available GeneAtlas database [41].

Of note, association between GW-significant SNPs and previously published SNPs was assessed by using SNiPA, an interactive and genetic variant annotation browser [42].

## Results

### Study demographics

Demographic and clinical characteristics of the PsyMetab cohort are reported in Table 1. One thousand one hundred and thirty-five patients who met the eligibility criteria and provided their written informed consent to participate in the present study were included. Median age was 46 years (IQR = 31−59 years), less than half of the patients were men (*n* = 520, 46%) and 42% of patients smoked. Patients were equally distributed between diagnoses of psychotic disorders (*n* = 196, 17%), bipolar disorders (*n* = 181, 16%) and depressive disorder (*n* = 191, 17%). Quetiapine was the most frequently prescribed psychotropic drug (*n* = 354, 31%). The majority (62%) of the patients received a psychotropic drug with a moderate propensity for inducing weight gain, while 217 patients (19%) received a psychotropic drug associated with a high risk of weight gain. In the whole cohort, the median baseline (i.e. before starting psychotropic treatment) BMI was 24.4 kg/m^2^, which is close to the definition of overweight (≥ 25 kg/m^2^).

**Table 1:** Clinical parameters of PsyMetab patients.

	*n* = 1135
**Age, median (IQR), years**	46 (31–59)
**Men,** ***n*(%)**	520 (45.8)
**Smoking status,** ***n*(%)**	482 (42.5)
**Diagnosis,** ***n*(%)**	
Psychotic disorders (F20-F24; F28-F29)	196 (17.2)
Schizoaffective disorders (F25)	57 (5.0)
Bipolar disorders (F30-F31)	181 (15.9)
Depressive disorders (F32-F33)	191 (16.8)
Organic disorders (F00-F09)	53 (6.4)
Other	156 (13.7)
Not available	301 (26.5)
**Psychotropic treatment,** ***n*(%)**	
Amisulpride	35 (3.1)
Aripiprazole	118 (10.4)
Clozapine	74 (6.5)
Mirtazapine	100 (8.8)
Olanzapine	123 (10.8)
Other^1^	113 (9.9)
Quetiapine	354 (31.2)
Risperidone	148 (13)
Valproate	70 (6.2)
Low metabolic risk^2^	211 (18.6)
Moderate metabolic risk^2^	707 (62.3)
High metabolic risk^2^	217 (19.1)
**Baseline BMI, median (IQR), kg/m**^**2**^	24.4 (21.4–28.1)

^1^Patients taking infrequently prescribed drugs (i.e., amitriptyline, levomepromazine, lithium, trimipramine, zuclopenthixol, carbamazepine, chlorprothixene, flupentixol, haloperidol, lurasidone and triapride) were considered in the “other” category.

^2^Subgroups were defined based on drug of follow-up. As participants could be followed for more than one drug, follow-ups were selected according to the priority drug list shown in the psychotropic treatment section. It is noteworthy that, among the drugs prescribed to patients in the present study, olanzapine, clozapine and valproate were considered in the high weight gain-inducer group, whereas amitriptyline, levomepromazine, lithium, mirtazapine, paliperidone, risperidone, quetiapine, trimipramine and zuclopenthixol were considered in the median weight gain-inducer groups and amisulpride, aripiprazole, carbamazepine, chlorprothixene, flupentixol, haloperidol, lurasidone and tiapride were considered in the low weight gain-inducer group.

### GWAS results in the PsyMetab cohort

Four loci were associated with BMI phenotypes in the PsyMetab cohort at genome-wide significance (*p* < 5 × 10^−8^). In particular, rs7736552 (near *MAN2A1*), rs11074029 (in *SLCO3A1*), rs117496040 (near *DEFB1*) and rs7647863 (in *IQSEC1*) were associated with BMI slope, BMI slope at 6 months, BMI change and BMI change at 3 months, respectively (Table 2; Supplementary Fig. 2). Of note that, although not GW significant, associations between these 4 loci and all alternative BMI phenotypes showed consistent effect directions (Supplementary Table 3). In addition, further analyses considering BMI in mixed effect models showed consistent findings and highlighted the importance of the genetic effects in particular during the initial six-month-period upon treatment start (Supplementary Table 3).

**Table 2:** GWAS-significant SNPs associated with BMI phenotypes in PsyMetab.

BMI phenotype	SNP	Nearest gene(s)	Gene location	Chromosome	Position (GRCh37)	Reference allele	Alternative allele	*N*	MAF	BETA	*p*-value
BMI slope	rs7736552	*MAN2A1, PGAM5P1, TMEM232*	Intergenic	5	109368613	A	G	1135	0.34	−0.2629	3.12E-08
BMI slope (6 months)	rs11074029	*SLCO3A1, LOC107984747*	Intron	15	92531950	C	T	985	0.25	0.2808	3.03E-08
BMI change	rs117496040	*DEFB1*	Intergenic	8	6743346	C	T	1135	0.01	1.1236	4.22E-08
BMI change (3 months)	rs7647863	*IQSEC1*	Intron	3	13289938	A	G	869	0.10	−0.4543	1.40E-08

*BMI* Body mass index, *GRCh37* Genome Reference Consortium Human Build 37, *MAF* Minor allele frequency, *SNP* Single nucleotide polymorphism.

**Fig. 1:**
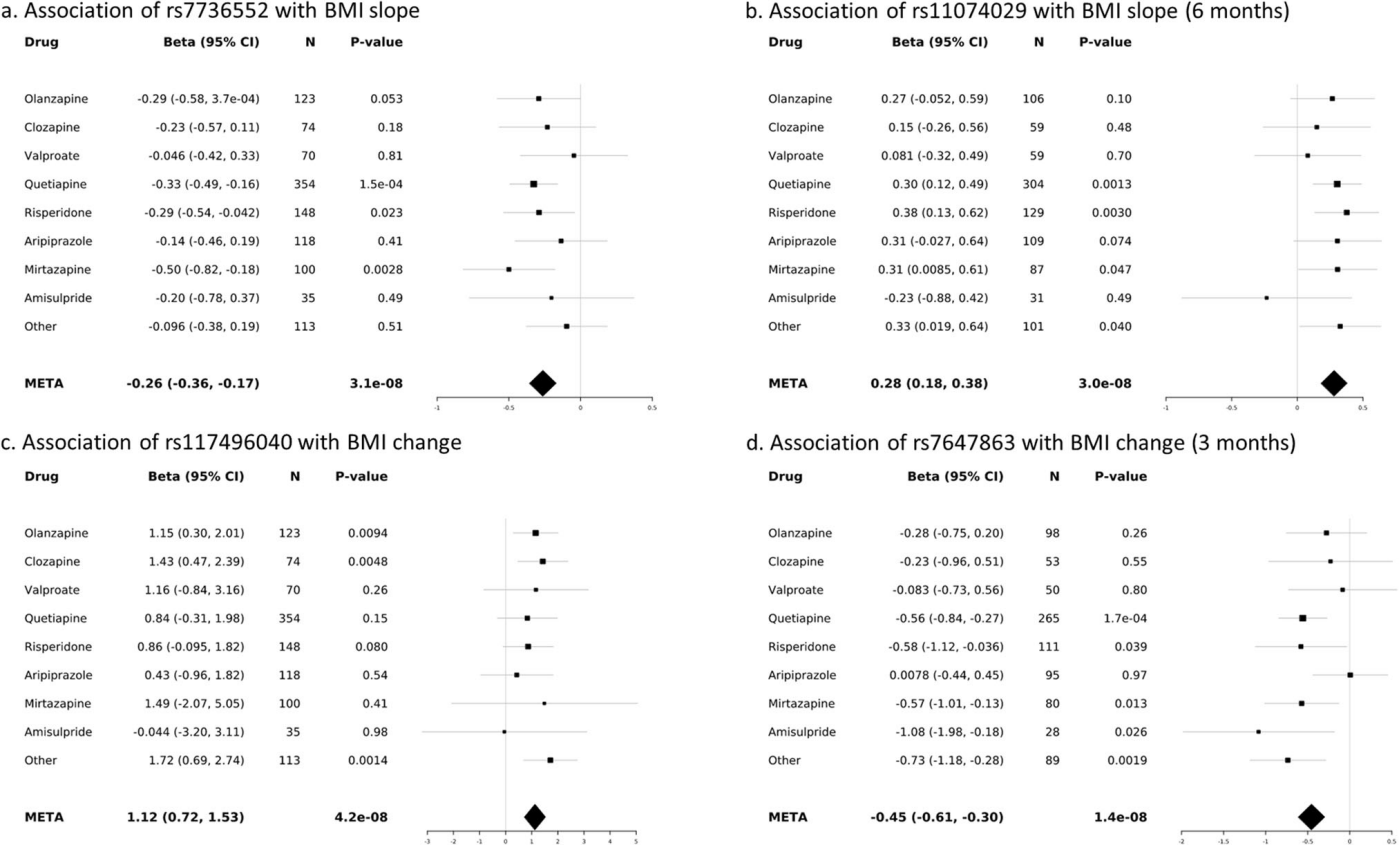
Forest plots of SNP association with metabolic phenotypes in PSYMETAB. **a** Association of rs7736552 with BMI slope; **b** Association of rs11074029 with BMI slope (6 months); **c** Association of rs117496040 with BMI change; **d** Association of rs7647863 with BMI change (3 months). Black squares and grey lines indicate beta and 95% confidence intervals for each drug, respectively. Black diamonds represent meta-analysis results. CI confidence interval, META meta-analysis.

Although the four loci showed no significant evidence of heterogeneity across psychotropic drugs (Cochran’s Q statistic *P*-values > 0.05), individual inspection of the results revealed that depending on the drugs, stronger, weaker or no associations were observed (Fig. 1).

### Replication of the four novel PsyMetab weight-gain associated loci in the UK Biobank (UKB)

Analyses conducted in UK Biobank participants diagnosed with a mental disorder and/or taking a psychotropic drug revealed a consistent association between rs7736552 and BMI slope (*p* = 0.017; Table 3), whereas no association was observed between the three remaining loci and BMI slope.

**Table 3:** Association between the four GWAS-significant SNPs and BMI slope in psychiatric subjects from the UK Biobank.

SNP	Nearest gene(s)	Gene location	Chromosome	Position (GRCh37)	Reference allele	Alternative allele	*N*	BETA	*p*-value
rs7736552	*MAN2A1, PGAM5P1, TMEM232*	Intergenic	5	109368613	A	G	1622	−0.088	**0.017**
rs11074029	*SLCO3A1, LOC107984747*	Intron	15	92531950	C	T	1622	0.03	0.43
rs117496040	*DEFB1*	Intergenic	8	6743346	C	T	1622	0.033	0.83
rs7647863	*IQSEC1*	Intron	3	13289938	A	G	1622	−0.047	0.47

Among the selected 1622 UK Biobank psychiatric subjects, the majority (*n* = 1470) are diagnosed with a psychiatric disorder and take psychotropic drugs, while the minority is taking a psychotropic drug but not diagnosed with a psychiatric disorder (*n* = 152).

Significant *p*-value is indicated in bold.

In 29,376 population-based (non-psychiatric) participants of the UKB, the four loci were not associated with BMI slope (*p* ≥ 0.16).

### Association of the four GWAS-significant loci with metabolic and psychiatric outcomes in consortia and in GeneAtlas

The four GW-significant SNPs were examined for association with metabolic and psychiatric phenotypes in relevant consortia and in the GeneATLAS database. Some nominal associations were observed (*p* < 0.05), namely rs7736552 (negatively associated with BMI slope in PSYMETAB at GW-significance) was also negatively associated with BMI in GIANT (*p* = 0.008; Supplementary Table 4). The risk allele of rs11074029 (positively associated with BMI slope restricted to 6 months in PSYMETAB) was significantly associated with a decreased risk of suffering from major mood disorders in PGC (*p* = 0.002; Supplementary Table 4). In the GeneAtlas database, this SNP was also negatively significantly associated with “psychological /psychiatric problem” (*p* = 0.0005), “bipolar affective disorder” (*p* = 0.01), “depression” (*p* = 0.01), “mania/bipolar disorder/manic depression” (*p* = 0.02) and was positively associated with “trunk fat-free mass” (*p* = 0.009) and whole body fat-free mass (0.02); Supplementary Table 5). The risk allele of rs117496040 (positively associated with BMI change in PsyMetab) was positively associated with “Schizophrenia, schizotypical and delusional disorders” in GeneAtlas (*p* = 0.04; Supplementary Table 5). Finally, the protective allele of rs7647863 (negatively associated with BMI change after 3 months of treatment in PsyMetab) was also negatively associated with BMI, T2D and fasting glucose in GIANT (*p* = 0.006), DIAGRAM (*p* = 0.03) and MAGIC (*p* = 0.02), respectively (Supplementary Table 4). In GeneAtlas, this SNP was also negatively associated with “hypertension” (*p* = 0.0002), “BMI” (*p* = 0.02), “unspecified diabetes mellitus” (*p* = 0.02), “non-insulin-dependent diabetes mellitus” (*p* = 0.02), “hypertensive diseases” (*p* = 0.02) and “essential (primary) hypertension” (*p* = 0.03), whereas it was positively associated with “impedance of whole body” (*p* = 0.008) (Supplementary Table 5).

## Discussion

To our knowledge, this is the largest GWAS investigating the evolution of BMI during psychotropic treatment to date. Four novel loci associated with BMI phenotypes were identified. SNP rs7736552, located near the *MAN2A1* gene, was associated with BMI slope during psychotropic treatment. This result was consistently replicated in UKB individuals suffering from a mental disorder as well as in the GIANT consortium. Mechanisms underlying these findings need to be further evaluated as nearest genes of this intergenic SNP (i.e. *MAN2A1*, *PGAM5P1* and *TMEM232*) have never been recognized to play a role in metabolic regulation. Moreover this SNP is not known to be associated with the expression levels of any gene.

The second loci identified in this study, rs11074029, located in the gene coding for the *Solute Carrier Organic Anion Transporter Family Member 3A1* (*SLCO3A1)*, was associated with BMI slope within 6 months following the introduction of psychotropic treatment. In agreement with this finding, previous studies reported associations between other SNPs in *SLCO3A1* (although not in linkage disequilibrium (LD) with rs11074029 (r^2^ < 0.8)) and BMI in various Caucasian population-based samples [43–45]. SLCO3A1 protein is involved in the transport of many molecules including glucose and other sugars, which may tentatively explain the observed associations with BMI phenotypes [46].

Intergenic SNP, rs117496040 located near the *DEFB1* gene, was associated with BMI change during psychotropic treatment in our psychiatric cohort. This gene (referred to as defensin beta 1), belongs to the family of microbicidal and cytotoxic peptides made by neutrophils. Interestingly, rs117496040 has been associated with an increased *DEFB1* expression in human adipose tissue [47] and a previous study observed differential expression of this gene in kidneys of diabetic-obese versus diabetic-lean rodents [48]. These findings suggest the interminglement of the immune (inflammation) and metabolic (obesity) systems [49].

Finally, rs7647863 localized in the *IQ Motif And Sec7 Domain ArfGEF 1 (IQSEC1)* gene, was associated with BMI change within 3 months following the introduction of psychotropic treatment. This result is in accordance with findings from other consortia, where significant associations between the SNP and BMI, T2D, fasting glucose (in GIANT, DIAGRAM and MAGIC consortia, respectively), hypertension, BMI and diabetes (according to GeneAtlas database) were observed. Considering that previous studies also found consistent associations between *IQSEC1* SNPs (not in LD (r^2^ < 0.8) with rs7647863) and BMI in population-based samples [43–45], this genetic region may potentially play a role in the regulation of metabolic features.

When the association between these SNPs and BMI was assessed cross-sectionally in large population-based cohorts (GIANT), two SNPs (rs7736552 and rs7647863) replicated in a directionally consistent manner at a nominally significance level. However, none of the four above-mentioned novel loci were associated with BMI slope in the UKB population-based cohort. This lack of association may be explained by the fact that the BMI phenotype used is a suboptimal measure of BMI evolution: BMI slope was constructed using two BMI estimates measured at heterogeneous time points, between which no known weight gain-inducing risk factor was introduced. Furthermore, signals of possible genetic susceptibilities for metabolic features may have been hindered in part by the fact that BMI slope considers equally obese and lean individuals whose weight is stable during the considered period.

Interestingly and despite the above-mentioned weaknesses of the use of BMI slope in the UKB cohort, rs7736552 was significantly associated with BMI slope in the UKB sample with a mental disorder diagnosis and with weight gain inducing psychotropic drugs. These results can be explained by the fact that individuals who suffer from mental disorders are more prone to develop weight gain than individuals from the general population, owing to the prescription of obesogenic psychotropic drugs but also to other factors including limited access to general somatic care and/or unhealthy lifestyle choices (e.g., physical inactivity, poor diet and high rates of cigarette smoking). In this way, greater influences of certain genetic variants on metabolic features have been reported in patients suffering from mental disorders as compared to the general population [18, 50–52]. Of note, the important contribution of some genetic markers on metabolic side effects induced by psychotropic drugs may be explained by the hypothesis that psychotropic drugs, that are very recent in the scale of human evolutionary history and applied to only a small fraction of the population, may have not yet been subject to strong natural selection, i.e., the frequency of associated genetic markers have not been strongly influenced.

Interestingly, rs11074029 and rs117496040 displayed significant associations with BMI and psychiatric phenotypes, in agreement with a recent GWAS reporting extensive polygenic overlap between BMI and major mental disorders which emphasized the complex interplay of metabolism-related gene pathways in the pathophysiology of mental disorders [53].

Results of the present study should be considered with the following limitations. Most patients included in the present study were not drug naïve, and the observed elevated baseline BMI may have resulted from previous psychotropic drugs. Nevertheless, this reflects the real psychiatric population observed in clinical practice and, despite the elevated median of baseline BMI, the present study had sufficient power to detect genetic hits significantly associated with BMI increase during the current psychotropic treatment. In the present study, about one third (i.e., 28%) of patients received multiple psychotropic drugs with different mechanisms of action at the same time, which may have introduced heterogeneity in the genetic associations. However, outcomes of interest (i.e., BMI modifications) were calculated from the initiation of the last introduced psychotropic drug to capture as best as possible the effect of the single last introduced drug. Although drug dosage of some psychotropic drugs have been observed to be associated with weight gain, the clinical effect is generally modest. In the present study, analyses restricted on single psychotropic drugs showed no significant correlation between drug dosage and weight gain. Of note, the consideration of additional cardiometabolic risk factors such as the waist-to-height ratio, waist-to-hip ratio or body fat mass would also have been valuable. However, in the present study, these data were recorded only partially and the power to detect genetic variants associated with the deterioration of the above-mentioned cardiometabolic variables would have been insufficient. Genetic studies considering the evolution of other cardiometabolic parameters during treatment with psychotropic drugs than BMI should therefore be conducted in the future. It is noteworthy that the use of the UKB cohort as a replication sample was not ideal due to its marked differences to PsyMetab (e.g., in terms of study design and phenotype information). For instance, psychotropic drug duration was not available in the UKB cohort and BMI estimates were measured independently of psychotropic drug initiation, which impeded appropriate assessment of psychotropic drug side-effects in this cohort. In addition, although the PsyMetab cohort was large, the number of participants on drugs with a high propensity of weight gain (e.g. olanzapine and clozapine) was relatively small. Larger studies (*n* > 1000) focused on single psychotropic drugs separately may allow for the identification of specific loci that could provide insights on drug-specific mechanisms underlying metabolic disturbances. Given the small study size and low number of genome-wide significant hits, we lacked the statistical power for various follow-up analyses (such as pathway analysis, tissue-enrichment analysis, Mendelian randomization, genetic correlation, etc.).

Strengths of the present study include its large sample size of such a particular sample. To our knowledge this is the largest GWAS investigating the evolution of BMI during psychotropic drug treatment. We analyzed six highly correlated BMI phenotypes in the present study, and despite the fact that the genome-wide significant findings were only identified in one phenotype, the four novel loci were consistently associated with the five remaining BMI phenotypes, emphasizing the robustness of our results. PsyMetab is a rich longitudinal data set, allowing proper phenotype correction of potentially confounding variables throughout psychotropic treatment. This cohort is very well positioned to analyze side-effects of psychotropic drugs as all participants have available baseline (i.e., at treatment initiation) data. It is noteworthy that at this stage the present results are preliminary. Predictive analyses in independent studies should be conducted before these results can be considered in clinical settings.

In conclusion, the present study identified four novel genetic loci associated with psychotropic-induced weight gain. These findings provide new insights into metabolic side effects induced by psychotropic drugs and underline the need for future studies to replicate these findings in larger cohorts. The combination of these results with epigenome-wide association studies (EWAS) and transcription studies would help further elucidate the mechanisms of psychotropic-induced metabolic disturbances and disentangle the role of the complex interplay between metabolic- psychiatric- and immune systems. Expanding phenotypes of interest to biomarker levels including total cholesterol, low-density lipoprotein cholesterol, high-density lipoprotein cholesterol, triglyceride, and glucose levels would also be of great interest for future research.

### Supplementary information

Supplementary Material
