# Supplementary material for: Identification of four novel loci associated with psychotropic drug-induced weight gain in a Swiss psychiatric longitudinal study: A GWAS analysis
Source: Mol Psychiatry. 2023 May 12;28(6):2320–7. doi: 10.1038/s41380-023-02082-3 (PMC10611564; doi:10.1038/s41380-023-02082-3)
Supplement: Supplementary file 1 — Supplementary Material [file 41380_2023_2082_MOESM1_ESM.docx]

**Supplementary Information**

**Methods**

## *Genotyping and quality control*

Ancestry was determined using *snpweights*, a software for inferring genome-wide (GW) ancestry using SNP weights precomputed from large external reference panels ^1^. For each participant, the software computes a respective percentage of European, East Asian, North American and East African ancestry. Using a threshold of 0.7 for East African, and 0.8 for all other ancestries, participants were attributed into one ethnic group. If no ancestry met this threshold, participants were defined as “mixed”. Principal component analysis of the genetically determined ancestries are displayed in **Supplementary Figure 1**.

Standard quality control (QC) filters were applied before imputation. First, missingness filters were applied at both the variant and individual level in an interactive process whereby variants with >10% missingness were removed first, followed by individuals with >10% missingness. This was repeated at 5% and 1% filters such that all variants and individuals had >99% completeness after filtering. Secondly, sex-check was applied to remove unambiguous sex violations. Finally, data was prepared for imputation according to Michigan Imputation Server guidelines and submitted to the server for imputation using HRC as a reference panel and European as the population ^2^.

## *Characterization of “psychiatric participants” in the UK Biobank*

UKB participants were selected to represent as best as possible the PsyMetab cohort, i.e. participants who receive a psychotropic drug or participants who have been diagnosed with a psychiatric disorder. Specifically, we selected participants who were taking at least one psychiatric drug matching those in PsyMetab (i.e. olanzapine, clozapine, valproate, quetiapine, risperidone, aripiprazole, mirtazapine or amisulpride) according to the drug use variable (variable 20003) termed UKB psychiatric drug users or who had been diagnosed with at least one psychiatric disorder (i.e. all diagnoses from “ICD10, Chapter V Mental and behavioral disorders”, see <https://biobank.ctsu.ox.ac.uk/showcase/field.cgi?id=41270>), termed “UKB ICD10 participants”.

### Supplementary Table 1: Psychotropic drugs included in the metabolic follow-up recommendation

| **ANTIPSYCHOTICS** | | **ANTIDEPRESSANTS** | | **MOOD STABILIZERS** |
| --- | --- | --- | --- | --- |
|  |  |  |  |  |
| **Atypical  (second-generation)** | **Typical  (first-generation)** | **Tricyclic** | **Other** |  |
|  |  |  |  |  |
| Amisulpride | Chlorprothixene | Amitriptyline | Mirtazapine | Carbamazepine |
| Aripiprazole | Flupentixol | Clomipramine |  | Lithium |
| Asenapine | Haloperidol | Doxepine |  | Valproate |
| Clozapine | Levomepromazine | Imipramine |  |  |
| Lurasidone | Pipamperone | Nortriptyline |  |  |
| Olanzapine | Promazine | Opipramol |  |  |
| Paliperidone | Sulpiride | Trimipramine |  |  |
| Quetiapine | Tiapride |  |  |  |
| Risperidone | Zuclopenthixol |  |  |  |
| Sertindole |  |  |  |  |
|  |  |  |  |  |

According to international recommendations, a metabolic follow-up is ongoing since 2007 in the Department of Psychiatry at the Lausanne University Hospital ^3^, in which inpatients and outpatients are prospectively monitored when starting a pharmacological treatment known to have a potential risk to induce metabolic disturbances (i.e. drugs listed above). The list is based on psychotropic drugs available in Switzerland). Of note, amisulpride, aripiprazole, clozapine, mirtazapine, olanzapine, quetiapine, risperidone and valproate represent 90% of drugs prescribed in patients included in the present study.

### Supplementary Table 2: summary of consortia used on PHEWAS analyses.

| **Trait** | **Consortia** | **Link** | **Description** | **Reference (PMID or DOI)** |
| --- | --- | --- | --- | --- |
| T2D (adjusted for BMI) | DIAGRAM | <https://diagram-consortium.org/downloads.html> | T2D GWAS meta-analysis - Adjusted for BMI | 30297969 ^4^ |
| T2D | DIAGRAM | <https://diagram-consortium.org/downloads.html> | T2D GWAS meta-analysis - Unadjusted for BMI | 30297969 ^4^ |
| BMI | GIANT | <https://portals.broadinstitute.org/collaboration/giant/index.php/GIANT_consortium_data_files> | GIANT and UK BioBank Meta-analysis | 30124842 ^5^ |
| Fasting glucose (BMI adjusted) | MAGIC | <https://www.magicinvestigators.org/downloads/> | Glucose results accounting for BMI are from an analysis of 29 studies in up to 58,074 non-diabetic participants and the insulin results accounting for BMI are from an analysis of 26 studies in up to 51,750 non-diabetic participants. | 22581228 ^6^ |
| Fasting glucose | MAGIC | <https://www.magicinvestigators.org/downloads/> | Glucose results accounting for BMI are from an analysis of 29 studies in up to 58,074 non-diabetic participants and the insulin results accounting for BMI are from an analysis of 26 studies in up to 51,750 non-diabetic participants. | 22581228 ^6^ |
| LDL | GLGC | <http://csg.sph.umich.edu/willer/public/lipids2013/> | LDL | 24097068 ^7^ |
| HDL | GLGC | <http://csg.sph.umich.edu/willer/public/lipids2013/> | HDL | 24097068 ^7^ |
| TG | GLGC | <http://csg.sph.umich.edu/willer/public/lipids2013/> | Triglycerides | 24097068 ^7^ |
| TC | GLGC | <http://csg.sph.umich.edu/willer/public/lipids2013/> | Total cholesterol | 24097068 ^7^ |
| BMI | GIANT | <https://portals.broadinstitute.org/collaboration/giant/index.php/GIANT_consortium_data_files> | BMI European ancestry | 25673413 ^8^ |
| Major depression disorder (MDD) | PGC | https://pgcdata.med.unc.edu/major_depressive_disorders/PGC_UKB_depression_genome-wide.txt | Depression, PGC combined with UKB | 30718901 ^9^ |
| Bipolar disorder | PGC | <https://pgcdata.med.unc.edu/bipolar_disorder/daner_PGC_BIP32b_mds7a_0416a.gz> | Bipolar GWAS | 31043756 ^10^ |
|  |  |  |  |  |

BMI: body mass index; DIAGRAM: DIAbetes Genetics Replication And Meta-analysis; GIANT: Genetic Investigation of Anthropometric Traits; GWAS: genome-wide association study; HDL: high-density lipoprotein cholesterol; LDL: low-density lipoprotein cholesterol; MAGIC: Meta-Analyses of Glucose and Insulin-related traits Consortium; PGC: Psychiatric Genomics Consortium; PHEWAS: phenome wide association studies; TC: total cholesterol; TG: triglyceride; T2D: Type 2 diabetes.

**Supplementary Table 3: Association of GWAS-significant SNPs with other BMI endpoints in PsyMetab**

Bold lines indicate GWAS significant results. Abbreviations: BMI: body mass index; GRCh37: Genome Reference Consortium Human Build 37; MAF: minor allele frequency; SNP: single nucleotide polymorphism. "BMI mixed" refers to mixed-effects models on BMI, for which p-value and estimate relate to the SNP x treatment duration interaction term.

**Supplementary Table 4: Association of SNPs of interest with metabolic or psychiatric phenotypes in consortia**

| **SNP** | **Consortium** | **Trait** | **Effect allele** | **Other allele** | **Beta** | **SE** | **P-value** |
| --- | --- | --- | --- | --- | --- | --- | --- |
| rs7736552 | GIANT | BMI | A | G | -0.005 | 0.0019 | 0.008 |
| rs11074029 | PGC | MDD | T | C | -0.015 | 0.005 | 0.002 |
| rs7647863 | GIANT | BMI | A | G | 0.009 | 0.0032 | 0.006 |
| rs7647863 | DIAGRAM | T2D (BMI adj) | A | G | 0.029 | 0.014 | 0.03 |
| rs7647863 | MAGIC | Fasting glucose | A | G | 0.016 | 0.0068 | 0.02 |

| BMI: Body Mass Index |
| --- |
| DIAGRAM: DIAbetes Genetics Replication And Meta-analysis |
| GIANT: Genetic Investigation of Anthropometric Traits |
| MAGIC: Meta-Analyses of Glucose and Insulin-related traits Consortium |
| MDD: Major Depression Disorder |
| PGC: Psychiatric Genomics Consortium |
| SE: Standard Error |
| T2D (BMI adj): Type 2 Diabetes, adjusted for Body Mass Index |

**Supplementary Table 5: Association of SNPs of interest with metabolic or psychiatric phenotypes in GeneAtlas database**

| **SNP** | **Trait** | **Effect  allele** | **Other  allele** | **Beta** | **P-value** |
| --- | --- | --- | --- | --- | --- |
|  |  |  |  |  |  |
| rs11074029 | Psychological / psychiatric problem | T | C | -0.002 | 0.0005 |
| rs11074029 | Trunk fat-free mass | T | C | 0.01 | 0.009 |
| rs11074029 | F31 bipolar affective disorder | T | C | -0.0003 | 0.01 |
| rs11074029 | Depression | T | C | -0.001 | 0.01 |
| rs11074029 | Mania / bipolar disorder / manic depression | T | C | -0.0003 | 0.02 |
| rs11074029 | Whole body fat-free mass | T | C | 0.02 | 0.02 |
|  |  |  |  |  |  |
| rs117496040 | F20-F29 Schizophrenia, schizotypal and delusional disorders | T | C | 0.00092 | 0.04 |
|  |  |  |  |  |  |
| rs7647863 | Hypertension | G | A | -0.005 | 0.0002 |
| rs7647863 | Impedance of whole body | G | A | 0.5 | 0.008 |
| rs7647863 | Body mass index | G | A | -0.03 | 0.02 |
| rs7647863 | Unspecified diabetes mellitus | G | A | -0.0007 | 0.02 |
| rs7647863 | Non-insulin-dependent diabetes mellitus | G | A | -0.002 | 0.02 |
| rs7647863 | Hypertensive diseases | G | A | -0.003 | 0.02 |
| rs7647863 | Essentiel (primary) hypertension | G | A | -0.003 | 0.03 |

### Supplementary Figure 1: Principal component analysis in PSYMETAB


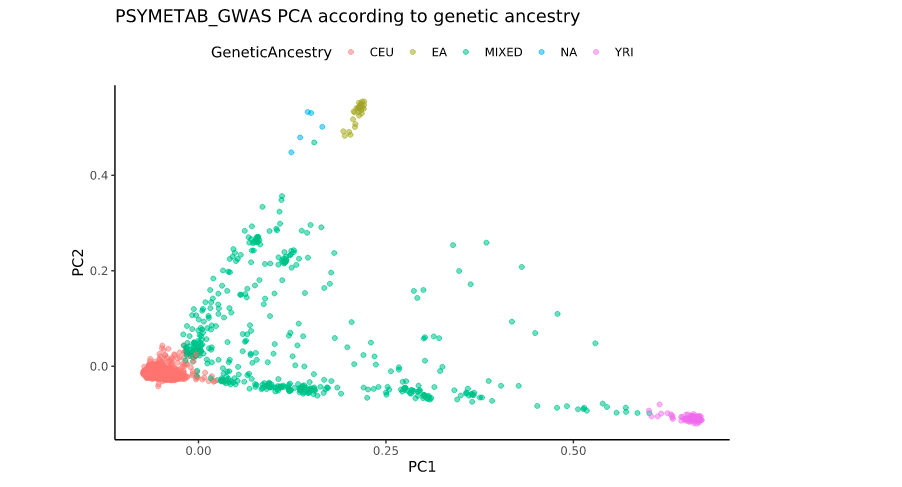


Figure shows principal component (PC) 1 vs PC2 color coded by reported ancestry (A) and genetic ancestry, as determined by snpweights software.

Abbreviations: CEU: Caucasian European, EA: East Asian, NA: North American, YRI: East African

### Supplementary Figure 2: Manhattan plots of BMI phenotypes in PsyMetab

1. BMI slope


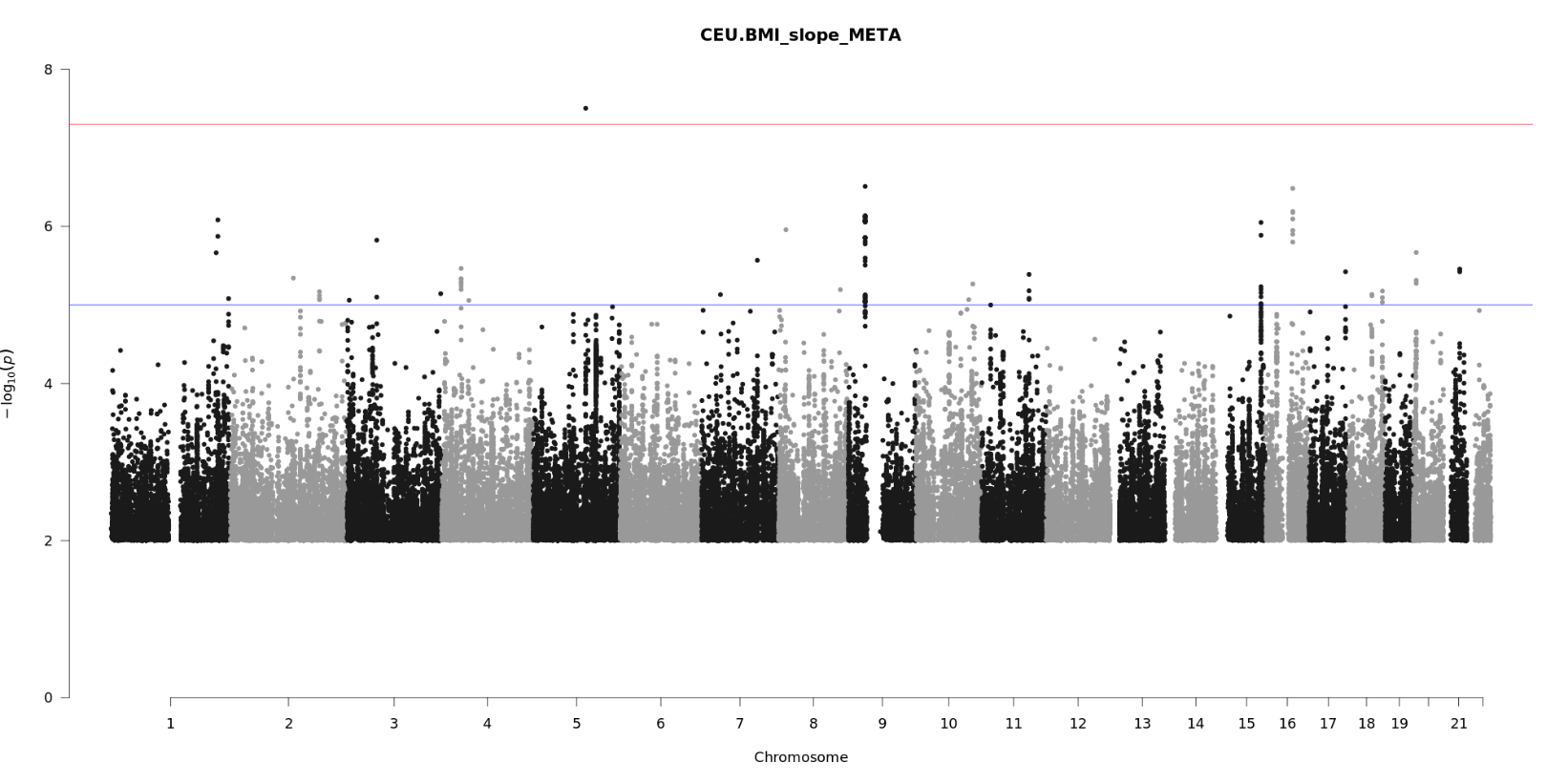


1. BMI slope (6 months)


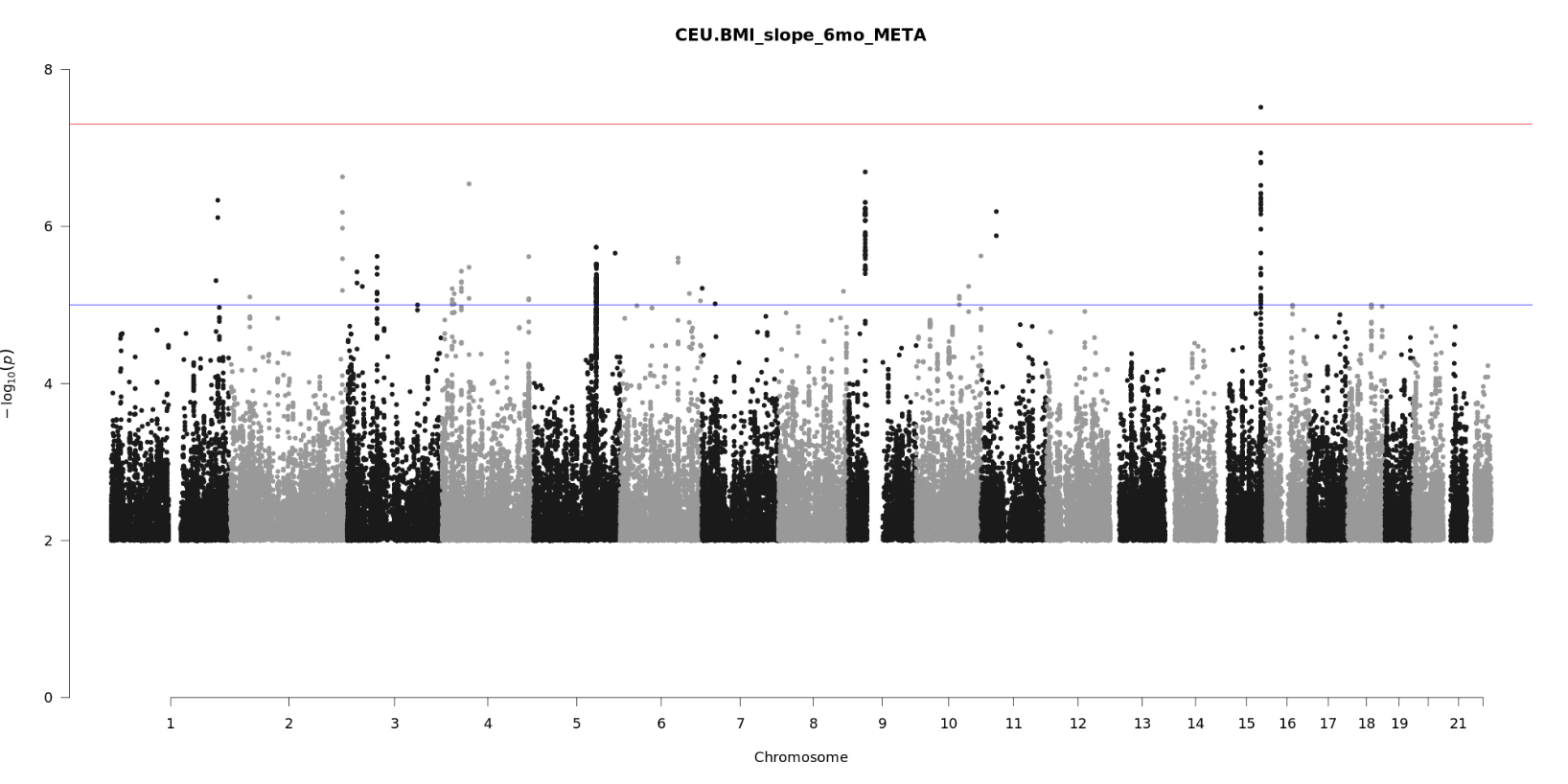


1. BMI change


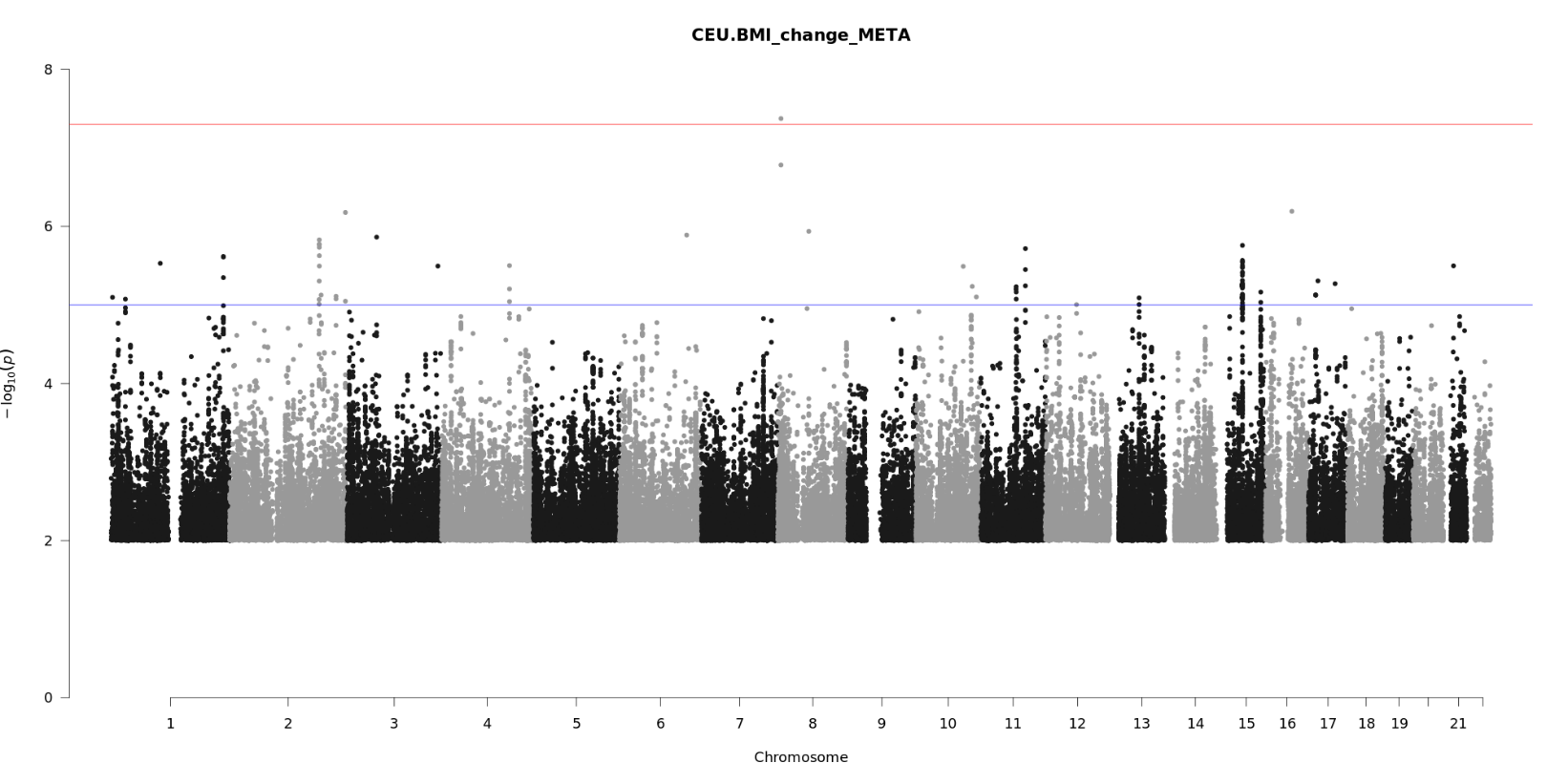


1. BMI change (3 months)


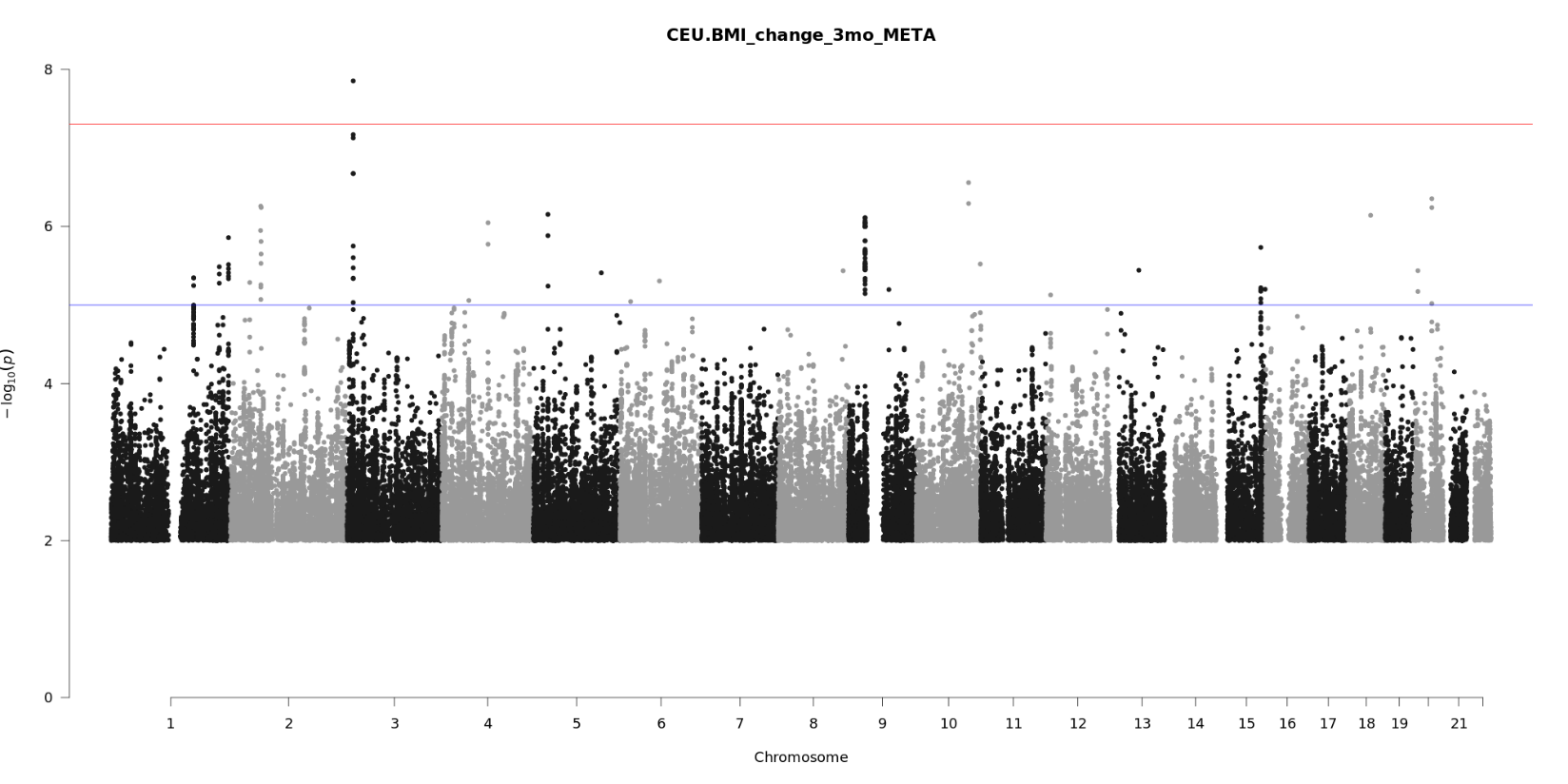


a. rs7736552 (near *MAN2A1*) is associated with BMI slope (p=3.12E-08);

b. rs11074029 (in *SLCO3A1*) is associated with BMI slope at 6 months (p=3.03E-08); c. rs117496040 (near *DEFB1*) is associated with BMI change (4.22E-08);

d. rs7647863 (in *IQSEC1*) is associated with BMI change at 3 months (p=1.40E-08)

### References

1. Chen CY, Pollack S, Hunter DJ, Hirschhorn JN, Kraft P, Price AL. Improved ancestry inference using weights from external reference panels. *Bioinformatics.* 2013;29(11):1399-1406.

2. Das S, Forer L, Schönherr S, et al. Next-generation genotype imputation service and methods. *Nature genetics.* 2016;48(10):1284-1287.

3. Choong E, Solida A, Lechaire C, Conus P, Eap CB. Follow-up of the metabolic syndrome induced by atypical antipsychotics: recommendations and pharmacogenetics perspectives. *Revue medicale suisse.* 2008;4(171):1994-1999.

4. Mahajan A, Taliun D, Thurner M, et al. Fine-mapping type 2 diabetes loci to single-variant resolution using high-density imputation and islet-specific epigenome maps. *Nature genetics.* 2018;50(11):1505-1513.

5. Yengo L, Sidorenko J, Kemper KE, et al. Meta-analysis of genome-wide association studies for height and body mass index in ∼700000 individuals of European ancestry. *Human molecular genetics.* 2018;27(20):3641-3649.

6. Manning AK, Hivert MF, Scott RA, et al. A genome-wide approach accounting for body mass index identifies genetic variants influencing fasting glycemic traits and insulin resistance. *Nature genetics.* 2012;44(6):659-669.

7. Willer CJ, Schmidt EM, Sengupta S, et al. Discovery and refinement of loci associated with lipid levels. *Nature genetics.* 2013;45(11):1274-1283.

8. Locke AE, Kahali B, Berndt SI, et al. Genetic studies of body mass index yield new insights for obesity biology. *Nature.* 2015;518(7538):197-206.

9. Howard DM, Adams MJ, Clarke TK, et al. Genome-wide meta-analysis of depression identifies 102 independent variants and highlights the importance of the prefrontal brain regions. *Nature neuroscience.* 2019;22(3):343-352.

10. Stahl EA, Breen G, Forstner AJ, et al. Genome-wide association study identifies 30 loci associated with bipolar disorder. *Nature genetics.* 2019;51(5):793-803.
